# Effects of Flavanols on Enteroendocrine Secretion

**DOI:** 10.3390/biom10060844

**Published:** 2020-06-01

**Authors:** Carme Grau-Bové, Carlos González-Quilen, Ximena Terra, M. Teresa Blay, Raul Beltrán-Debón, Rosa Jorba-Martín, Beatriz Espina, Montserrat Pinent, Anna Ardévol

**Affiliations:** 1 MoBioFood Research Group, Departament de Bioquímica i Biotecnologia, Universitat Rovira i Virgili, c/Marcel·lí Domingo nº1, 43007 Tarragona, Spain; carme.grau@urv.cat (C.G.-B.); carlosalberto.gonzalez@urv.cat (C.G.-Q.); ximena.terra@urv.cat (X.T.); mteresa.blay@urv.cat (M.T.B.); raul.beltran@urv.cat (R.B.-D.); anna.ardevol@urv.cat (A.A.); 2 Institut d’Investigació Sanitària Pere Virgili (IISPV), 43005 Tarragona, Spain; rosa.jorba1@gmail.com (R.J.-M.); bespina84@gmail.com (B.E.); 3 Servei de Cirurgia General i de l’Aparell Digestiu, Hospital Universitari Joan XXIII, 43005 Tarragona, Spain

**Keywords:** GLP-1, PYY, CCK, intestine, flavonoids

## Abstract

Some beneficial effects of grape seed proanthocyanidin extract (GSPE) can be explained by the modulation of enterohormone secretion. As GSPE comprises a combination of different molecules, the pure compounds that cause these effects need to be elucidated. The enterohormones and chemoreceptors present in the gastrointestinal tract differ between species, so if humans are to gain beneficial effects, species closer to humans—and humans themselves—must be used. We demonstrate that 100 mg/L of GSPE stimulates peptide YY (PYY) release, but not glucagon-like peptide 1 (GLP-1) release in the human colon. We used a pig ex vivo system that differentiates between apical and basolateral intestinal sides to analyse how apical stimulation with GSPE and its pure compounds affects the gastrointestinal tract. In pigs, apical GSPE treatment stimulates the basolateral release of PYY in the duodenum and colon and that of GLP-1 in the ascending, but not the descending colon. In the duodenum, luminal stimulation with procyanidin dimer B2 increased PYY secretion, but not CCK secretion, while catechin monomers (catechin/epicatechin) significantly increased CCK release, but not PYY release. The differential effects of GSPE and its pure compounds on enterohormone release at the same intestinal segment suggest that they act through chemosensors located apically and unevenly distributed along the gastrointestinal tract.

## 1. Introduction

The gastrointestinal tract is in charge of nutrient digestion and absorption. In addition, it is one of the bigger hormonal tissues. It is a source of various regulatory peptide hormones, secreted along the gastrointestinal tract by different enteroendocrine cells, which are involved in the coordination of digestive processes within the gastrointestinal system via autocrine and paracrine effects. Gut peptides, acting as both hormones and neurotransmitters, allow signalling between the periphery and central nervous system to coordinate systemic changes in our physiology [1]. The presence of enterohormones along the gastrointestinal tract is uneven and differs between species [2]. Among these enterohormones are found cholecystokinin (CCK), glucagon-like peptide 1 (GLP-1), and peptide YY (PYY). CCK acts as an anorexigenic peptide, inducing a decrease in food intake and body weight and an increase in perception of fullness, as well as regulating gastric emptying, gall bladder contraction, and pancreatic enzyme release [3,4]. PYY is often co-expressed and secreted with GLP-1 into the circulation in response to food intake, and it has been involved in energy homeostasis by regulating food intake and suppressing excessive consumption [5,6,7]. GLP-1 increases glucose-dependent insulin release; reduces glucagon secretion, thereby contributing to limiting postprandial glucose excursions; and decreases gastric emptying [8,9]. Altogether, considering the importance of these hormones on regulation of body homeostasis, modulation of the enteroendocrine system has become a target for treatment of obesity and type 2 diabetes [10,11].

Grape seed proanthocyanidin extract (GSPE) is a source of flavan-3-ols compounds including catechin and epicatechin monomers and their respective oligomers. GSPE has been shown to be beneficial against obesity and the metabolic syndrome [12,13]. Suggested mechanisms used by GSPE to exert this effect include modulation of the enteroendocrine system [14]. GSPE is a mixture of compounds, some of which are not absorbed and pass through the intestine to reach the colon, where they undergo metabolization [15]. In rats, a model in which polyphenols can be more easily measured, catechin, epicatechin, and dimmer B2 were found along the intestine after GSPE administration [16]. Unmetabolyzed flavan-3-ols (catechin, epicatechin or oligomers) have been detected in feces in pigs [17] and humans [18]. Thus, not only the polyphenol’s metabolites, but also some of the original compounds found in GSPE may interact with the enteroendocrine cells that are distributed along the gastrointestinal tract. In rats, acute GSPE treatment inhibits food intake (this is partly mediated by an increase in GLP-1 levels [19,20]), while ex vivo studies in rat intestinal segments have shown that GSPE directly stimulates GLP-1 and PYY release [21]. GSPE contains a mixture of different molecules and it has not yet been explained which of these are responsible for the effects of GSPE in stimulating enterohormone release. Studies in a ghrelin-secretory cell line have shown that the molecules that make up the grape extract have different effects on ghrelin secretion, for example, monomeric molecules stimulate ghrelin secretion through interaction with bitter taste receptors, while polymeric forms inhibit ghrelin secretion [22]. Several polyphenols have been shown to bind and activate bitter taste receptors, but those studies were conducted in silico [23] or were based on Ca^2+^ release in HEK293 cells that express human bitter taste receptors [24]. Therefore, although bitter taste receptors activate enterohormone release [25], the direct effects of polyphenols have not been tested for most of them. In vitro assays with STC-1 cell line have shown that flavonoids such as naringenin and hesperetin are able to stimulate CCK release [26,27]. Some other flavonoids as quercetin, kaempferol, and apigenin have also resulted in an increase in CCK levels in vitro, while others such as rutin and baicalein have not [28]. Moreover, in the STC-1 cell line, no stimulatory effect of epicatechin monomers, epicatechin gallate, procyanidin B2 dimer, or B2 gallate on GLP-1 or CCK was found [21]. In an ex vivo assay of murine intestines, epigallocatechin gallate stimulates CCK secretions in the duodenum [29], as well as GLP-1 secretions in the ileum [29]. Altogether, these results did not shed light on which molecules of those found in GSPE might contribute to its enterohormone releasing effects.

The above-mentioned studies were performed in STC-1 cells and ex vivo in intestinal tissue. The STC-1 cell line is shown to be suitable for studying enterohormone response to nutrients, though it has certain weaknesses [30], as cells are cultivated as monolayers on plastic surfaces and lack a normal cellular environment and polarization. Ex vivo studies maintain cellular environment. Furthermore, the distribution of enterohoromones and chemoreceptors that sense nutrients and activate the release of the hormones also differs along the intestine [31,32], and ex vivo studies offer the advantage over cell lines that the different intestinal segments can be tested. However, neither the cell lines nor the ex vivo studies enable luminal stimulation to be conducted. To overcome this problem, various ex-vivo approaches have been developed, including everted sacs, perfused intestinal loops, Ussing chambers [33], intestinal punches, precision-cut intestinal slices (PCIS) [34], organoids [35], and gut-on-a-chip [36].

In this paper, we used a specifically developed approach based on a porcine ex vivo system [37] to conduct a vectorial study of the effects of apically added bioactive compounds on the basolateral secretion of enterohormones. We used this system to investigate the effect of GSPE on PYY, GLP-1, and CCK secretion, and to identify some of the molecules responsible for this effect. To compare with our results in pig, in parallel, we also studied how treating human colon explants (a common ex vivo system) with GSPE affected the secretion of the same enterohormones.

## 2. Materials and Methods

### 2.1. Materials

GSPE was obtained from Les Dérivés Résiniques et Terpéniques (Dax, France). The same batch (#124029) was used in all studies. According to the manufacturer, the extract contains monomers of flavan-3-ols (21.3%), and dimeric (17.4%), trimeric (16.3%), tetrameric (13.3%), and oligomeric (5–13 U; 31.7%) proanthocyanidins. Catechin, (-)-Epicatechin, gallic acid (GA), 3-hydroxyphenyl acetic acid (3-HPAA), and protocatechuic acid (PCA) were obtained from Sigma (St. Louis, MO, USA), while procyanidin dimer B2 (B2) was obtained from Extrasynthese (Genay, France). For all studies, stocks were prepared in dimethyl sulfoxide (DMSO) and further diluted in the specific buffer required for each experiment. To transport and treat the intestinal samples, we used Krebs–Ringer bicarbonate (KRB) buffer (Hepes 11.5 mM, CaCl_2_ 2.6 mM, MgCl_2_ 1.2 mM, KCl 5.5 mM, NaCl 138 mM, NaHCO_3_ 4.2 mM, NaH_2_PO_4_ 1.2 mM) pH 7.4, prepared with either 10 mM D-Glucose (KRB-D-Glucose buffer) or 10 mM D-Mannitol (KRB-D-Mannitol buffer). For the enterohormone secretion studies, KRB-D-Glucose was supplemented with protease inhibitors: 10 µM amastatin (Enzo Life Sciences, Madrid, Spain), 500 KIU aprotinin (Sigma, Madrid, Spain), and 0.1% fatty acid free-bovine serum albumin.

### 2.2. Ex Vivo Pig Experiments

Pig intestines were obtained and mounted in the Ap-to-Bas ex vivo system as previously described [37]. Briefly, intestinal tissues were obtained from female pigs (*Sus scrofa domesticus*) sacrificed for meat production at a local slaughterhouse. The duodenum, ascending colon, and descending colon were collected for the experiments; transported to the laboratory in ice-cold KRB-D-Mannitol buffer saturated with 95% oxygen and 5% CO_2_; and immediately used for the ex vivo experiments. The time between excision and the beginning of the experiments was approximately 30 min.

Serosal and outer muscular layers were removed and circles of tissue with a diameter of 14 mm were punched out using a biopsy punch. The whole process was maintained at a low temperature with cold buffer and an ice-cold bath. Pieces of 1.5 cm in length were cut up and the apical side of the intestinal segment was glued (with 3M Vetbond, Cat No.: 1469SB, St. Paul, MN, USA) to a silicon tube, which was then placed inside a cell culture insert without a bottom membrane (Cat No.: MCRP12H48, 12-well hanging inserts). The entire insert containing the tissue segment and the piece of tube was placed in the well of a 12-well plate prefilled with 1 mL of KRB-D-Glucose buffer. Apically, the tube was filled with 400 μL of KRB-D-Mannitol buffer. The tissues were then pre-incubated at 37 °C for 15 min in a humidified incubator (5% (*v*/*v*) CO_2_).

Treatments were initiated by replacing basolateral KRB-D-Glucose with new KRB-D-Glucose with protease inhibitors and the apical KRB-D-Mannitol buffer solution with 400 μL of pre-warmed (37 °C) KRB-D-Glucose buffer containing the test compounds. KRB-D-Glucose buffer was used as a control. After 30 min of treatment, an aliquot of 200 μL was picked from the basolateral side of the Ap-to-Bas system. Finally, 60 min (for colon) or 90 min (for duodenum) after the beginning of the experiment, the entire volume of the apical and basolateral sides was frozen and stored at −80 °C for later analysis of the enterohormones.

### 2.3. Ex Vivo Human Colon Experiments

Human colon samples were obtained and processed as previously described [38] using an ex vivo system of human colon derived from the healthy margin of the mucosae near the fragment that is excised from colon cancer patients who require colectomy. These procedures were conducted at the Hospital Universitari Joan XXIII in Tarragona, Spain. The subjects were aged between 45 and 85. The exclusion criteria were as follows: alcohol intake above 30 g/day; body mass index above 40 kg/m^2^; use of drugs unrelated to metabolic syndrome treatment; the presence of intestinal malabsorptive or inflammatory bowel diseases; the presence of acute or chronic inflammatory or infectious disease; and the presence of neoplastic disease at advanced stages or requiring pharmacological treatment. Table 1 shows the characteristics of the subjects used for the study.

The samples were obtained during surgical treatment. Immediately after their extraction in the operating room, the samples were inserted into an ice-cold KRB-D-Mannitol buffer saturated with 95% oxygen and 5% CO_2_ and transferred to the laboratory within 20 min. There, the tissues were rinsed and the serosal and outer muscular layers were removed with a scalpel. After a 10 min washing period, tissue segments were placed in pre-warmed (37 °C) KRB-d-Glucose buffer 0.1% DMSO with protease inhibitors and either GSPE or vehicle. The samples were treated for 30 min in a humidified incubator at 37 °C and 5% CO_2_. Media were collected and frozen at −80 °C for enterohormone analysis. This experimental procedure was approved by the Clinical Research Ethics Committee (CEIC) of the Hospital Universitari Joan XXIII in Tarragona (CEIm 101/2017).

### 2.4. Enterohormone Quantification

The enterohormones were assayed using commercial ELISA kits in accordance with the manufacturer’s instructions: PYY from human and pig intestinal segments were measured using fluorescent immunoassay kits (Cat No.: FEK-059-02 and Cat No.: FEK-059-03, respectively, Phoenix Pharmaceuticals, Burlingame, CA, USA). CCK from pig samples was measured using an ELISA kit (Cat No.: EKE-069-04, Phoenix Pharmaceuticals, Burlingame, CA, USA). GLP-1 from both pig and human colon samples was measured using an ELISA kit (Cat No.: EZGLPT1-36k, Millipore, Burlington, MA, USA).

### 2.5. Statistical Analysis

The results are presented as mean ± SEM. Data were analysed using XLSTAT 2020.1 (Addinsoft, Barcelona, Spain) statistical software. Statistical differences were assessed by Student’s *t*-tests between the means of each treatment and the control. Significance was accepted over 5%.

## 3. Results

### 3.1. Apical Treatment with GSPE Stimulates Basolateral GLP-1 and PYY Secretion from Pig Intestinal Segments

**Figure 1:**
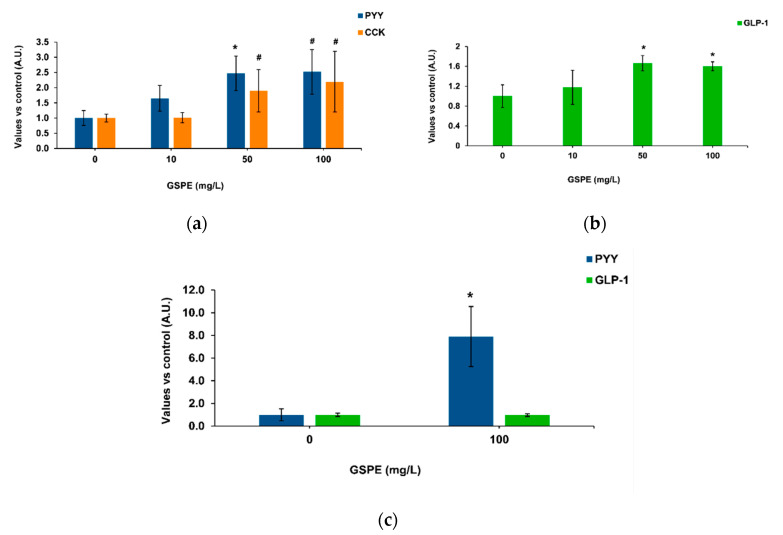
Enterohomone secretion levels in the basolateral medium after apical stimulation of pig intestinal segments with grape seed proanthocyanidin extract (GSPE). (**a**) Cholecystokinin (CCK) (orange columns) and peptide YY (PYY) (blue columns) secretion in duodenum. (**b**) Glucagon-like peptide 1 (GLP-1) secretion in ascending colon. (**c**) PYY (blue columns) and GLP-1 (green columns) secretion in descending colon. Results are expressed in arbitrary units (A.U.), which are values normalized versus the control and represent mean ± SEM. * *p* < 0.05 vs. control, # *p* < 0.1 *t*-test, n = 7–15.

Our methodology was based on intestinal pig samples mounted on a system that enables apical treatments and measures the basolateral secretion of enterohormones [37]. Figure 1a shows the effects of GSPE on pig duodenum, where 50 mg/L of GSPE stimulated PYY secretion. No significant effects were found on CCK secretion, but a tendency to an increase at the highest concentrations. In the duodenum, there was not a dose response effect of GSPE (analysis of variance (ANOVA) test, data not shown). In the ascending colon, an increase in basolateral GLP-1 secretion was observed with 50 and 100 mg/L GSPE (Figure 1b), again without a dose-response effect (ANOVA test, data not shown). In the descending colon, 100 mg/L GSPE did not modify GLP-1 secretion, but a strong increase in PYY was observed (Figure 1c). It has previously been shown in rat intestinal explants that higher doses do not affect tissue viability [21] or membrane permeability [38].

### 3.2. Apical B2 and Catechin/Epicatechin Specifically Stimulate PYY and CCK Secetion, Resectively

**Figure 2:**
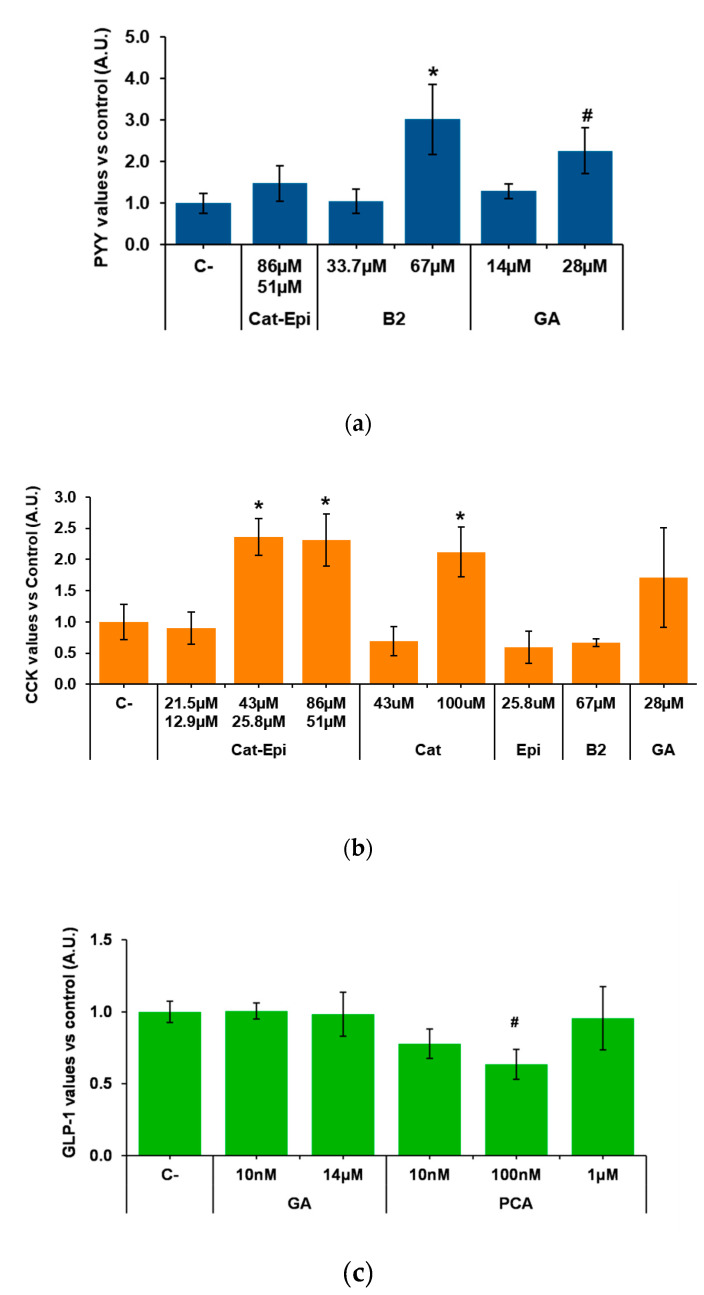
Enterohormone levels in the basolateral side after apical stimulation of pig intestinal samples with different purified compounds, for 60 min. (**a**) PYY secretion in the duodenum, (**b**) CCK secretion in the duodenum, (**c**) GLP-1 secretion in the ascending colon. Results are expressed in arbitrary units (A.U.), which are values normalized versus the control and represent mean ± SEM. * *p* < 0.05 vs. control, # *p* < 0.1, *t*-test. n = 7–9. C-, negative control vehicle-treated; Cat, catechin; Epi, epicatechin; B2, B2 procyanidin dimer; GA, gallic acid; PCA, protocatechuic acid.

We then analysed whether purified molecules contained in GSPE stimulate enterohormone release. To do so, we first tested, in the duodenum, the most available and abundant pure compounds of GSPE. According to the manufacturer, monomeric flavanols account for 21.3% of the phenolics in GSPE. A mixture of the monomers catechin plus epicatechin in the proportion in which it is found in GPSE [39] did not modify PYY secretion (Figure 2a). Gallic acid, a different monomeric form of phenolic compound that is highly abundant in GSPE, showed no effect at 14 µM. However, a tendency to increase PYY secretion was found at double the concentration. Dimeric structures represent 17.4% of the GSPE and, to represent them, we assayed dimer B2. When tested at a concentration equivalent to the content of dimers in 100 mg/L GSPE, B2 did not modify PYY secretion. However, doubling the dose to 67 µM significantly increased PYY secretion. In the descending colon, B2 also significantly increased PYY secretion (at 1.000 ± 0.04 and 1.300 ± 0.14 in control and B2, respectively; values normalized to control levels, *p* = 0.038, n = 11–13).

The effects of purified phenolic forms were also tested for CCK secretion in pig duodenum. We found that catechin/epicatechin (43/25.8 µM, respectively) induced CCK secretion. A twofold stimulation appeared to be the maximum achievable because, when double the dose (86/51.1 µM) was tested, the same level of stimulation was observed. Instead, a half-reduction in concentration did not induce CCK release (Figure 2b). The stimulatory effect of the mixture was not the result of just one of the monomers, because, when catechin and epicatechin were tested alone at the same concentration at which the mixture was effective, they did not induce CCK release. On the other hand, when only one monomer was tested at a concentration similar to the sum of both monomers in the mixture (e.g., catechin at 100 µM), CCK release significantly increased to an equivalent level (Figure 2b). Figure 2b also shows that gallic acid and B2 had no significant effects on pig duodenal CCK secretion.

Finally, samples from the descending colon were used to test the effects of gallic acid and protocatechuic acid (PCA), which is found in GSPE, but is also one major GSPE metabolite produced in the colon [40]. Figure 2c shows no effects of gallic acid or PCA on GLP-1, but a tendency towards a reduction by 100 nM PCA.

### 3.3. The Stimulation of Enterohormone Secretion by Purified Molecules Takes Place Apically

To confirm that the stimulatory effects of B2 on PYY and of catechins on CCK were not the result of the interaction between possibly absorbed forms and factors of the basolateral side of the intestine, we treated duodenal samples with B2 or epicatechin/catechin, in this case, administered at the basolateral side, and measured enterohormone release to the medium. We found no increase in basolateral PYY after 33.7 µM B2 treatment (1.000 ± 0.12 and 0.769 ± 0.25 in control and B2, respectively; values normalized to control levels, *p* = 0.39, n = 7–8). Similarly, basolateral administration of 43/25.8 µM epicatechin/catechin showed no significant effect on basolateral CCK concentration (1.000 ± 0.13, 0.774 ± 0.07 in control and catechin mixture, respectively; values normalized to control levels, *p* = 0.189, n = 6).

### 3.4. GSPE Stimlates PYY Secretion in Human Colon

**Figure 3:**
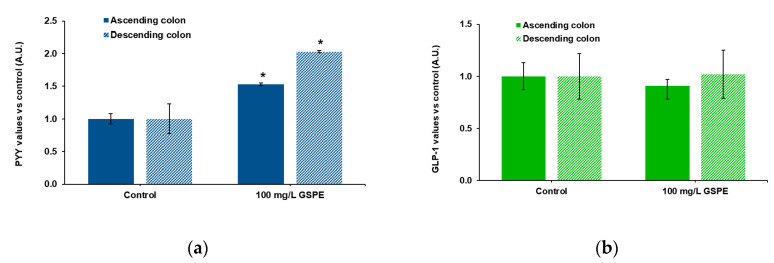
Enterohomone secretion in the medium after stimulation of human ascending (dark columns) and descending (light columns) colon explants with grape seed proanthocyanidin extract (GSPE, 100 mg/L). (**a**) PYY values, (**b**) total GLP-1 values. Results are expressed in arbitrary units (A.U.), which are values normalized versus the control and represent mean ± SEM. * *p* < 0.05 vs. control, *t*-test. n = 13–15 explants from 4–10 humans.

Finally, we checked the effects of GSPE on human intestine. These experiments were performed in explants because tissue availability did not enable us to mount the Ap-to-Bas system. Ascending and descending human colon explants were used to test the effects of GSPE on PYY and total GLP-1 secretion. Figure 3a shows that stimulation with 100 mg/L of GSPE led to a significant increase in PYY release to the medium in both intestinal segments. On the other hand, GSPE did not modify GLP-1 levels in the medium of either the ascending or the descending colon (Figure 3b).

## 4. Discussion

We have previously shown that a grape seed proanthocyanidin extract modulates enterohormone secretion in rat explants [21]. We now refine our system using an ex vivo model to separate the apical and basolateral side and use pig as a more similar model to human intestine than rat [41] in order to clarify the effects of the extract and some of its compounds along the gastrointestinal tract.

We first show that GSPE stimulates basolateral PYY and GLP-1 release in pig explants. In rats, the effects of GPSE on PYY were tested in the ileum [21]. As pigs express PYY in the duodenum and the descending colon [2], we tested PYY release in those tissues. Our results show that GSPE stimulates PYY release in the descending colon more than in the duodenum. Our data suggest that basal PYY secretion is lower in the colon than in the duodenum (Supplementary Materials, Table S1). According to Albrechtsen et al., pigs express PYY in the duodenum and the descending colon at the same level [2]. However, other studies show a greater gene expression of PYY in the colon than in the duodenum [31,42]. Similarly, we found significant GLP-1 release induced by GSPE in the ascending colon, but not in the descending colon; in the latter, however, we observed a higher GLP-1 basal secretion (Supplementary Materials, Table S1). Previous studies showed similar GLP-1 expression in the ascending and descending colon [2,42]. The basal enterohormone secretion we found may thus not reflect tissue content. In any case, GSPE does not act more effectively where there is a major expression. It is likely, therefore, that the various intestinal segments respond differently to GSPE stimulus not because of the amount of enterohormone in each tissue, but because of different chemosensory machinery. Nutrient receptors along the pig gastrointestinal tract are differently expressed [31,42]. A higher gene expression in the colon has been found for certain nutrient and non-nutrient receptors related to enterohormone release, such as the fatty acid transporter GPR120 [31,42,43], as well as for the cannabinoid receptor GPR119 [42]. It has been suggested that phenolic compounds bind bitter taste receptors (TAS2R) [44,45], but information on the expression of this family of receptors along the gastrointestinal tract is scarce [46].

The above results also showed that PYY secretion and GLP-1 secretion were not always equally stimulated. In the pig ascending colon, we found significant GLP-1 release, but, in agreement with a previously reported lack of PYY expression in the pig ascending colon [2], we were unable to detect stimulation of PYY release. In the descending colon, on the other hand, we found only stimulated PYY release. This could be because the proportion of cells containing each enterohormone is different. It has been shown in mice that roughly 15% of descending colon enteroendocrine cells contain GLP-1 alone and 7% contain PYY alone [47]. This is in line with our previous results in rat explants, where we found a stronger effect of digested GSPE on GLP-1 secretion [21]. In the pig colon, 40% of GLP-1/PYY containing cells contain GLP-1 alone and 7% contain PYY alone [47], though the above paper does not specify whether this is in the ascending or descending colon. In the human colon, almost all cells with GLP-1 also contain PYY and 25% of them contain PYY without GLP-1 [48]. In the present paper, we show that GSPE stimulated PYY release in the human colon. In this case, the effect is stronger where basal PYY secretion is higher, in the descending colon (Supplementary Materials, Table S1). However, we found no effects on GLP-1 at any of these locations. A lower number of only GLP-1-containing cells found in the human colon may thus explain why we did not detect any effects on GLP-1 secretion, but GSPE acts on PYY. However, in pig, we do not know enough about enterohormone co-expression to confirm this. Another possibility is that secretion may somehow be regulated so that a cell that produces and stores both hormones may still secrete mainly one of the products [6]. This hypothesis is supported by the observation that GLP-1 and PYY are stored separately in different vesicles in L cells [49,50]. Most studies that have analysed the secretion of GLP-1 and PYY show that of both these hormones are similarly stimulated by bioactive molecules [6,51]. However, there are exceptions. For example, in an pig ex vivo ileum model, sucrose was shown to stimulate GLP-1 secretion, but not PYY secretion, while other compounds stimulated the secretion of both hormones [51]. Perhaps GSPE differentially activates PYY and GLP-1.

One reason for the differential stimulation of co-stored enterohormones by GSPE could be that the various compounds in the mixture act differently in the hormone secretion.

**Figure 4:**
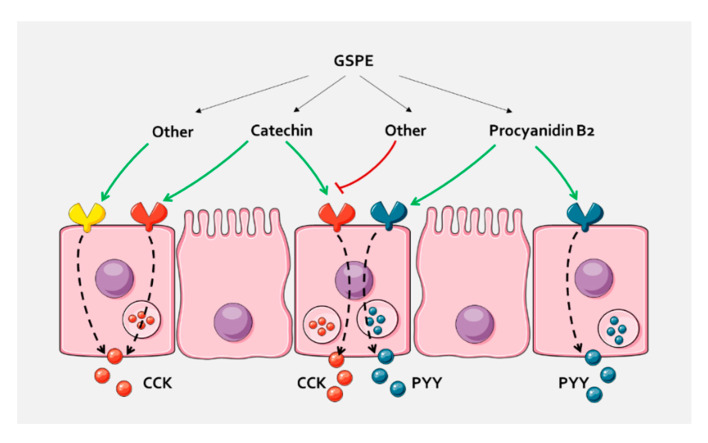
In pig duodenum, the different compounds of GSPE cause a selective enterohormone secretion. A possibility is that they act through different receptors that are related to a specific enterohormone secretion, either because they are expressed in cells containing only this hormone, or because they activate intracellular mechanisms that distinguish between hormones. Other compounds of the extract might antagonize the effects, or increase secretion through the same or other receptors. A similar pathway could take place in the colon, where differential GLP-1 and PYY secretion has been found.

We thus tested the effects of pure compounds that are abundant in GSPE. Our results show that, in pig duodenum, dimer B2 activates PYY release. Our ex vivo system enables us to study the effect on secretion in a more physiological situation than with explants [37]. It has been suggested that the absorption and metabolism of flavonoids, and particularly B2, are rare [52]. However, most studies suggest that flavonoids can be absorbed in the upper gastrointestinal tract [15]. In any case, in this paper, we show that B2 interacts with some pathway that is initiated on the apical side and rule out that the effects are mediated through direct interaction with enterohormone-releasing sensory systems on the basolateral side, such as calcium-sensing receptors [53]. To our knowledge, the in vivo enterohormone-secretory effect of B2 has not previously been demonstrated. This finding could help to explain previously found B2 effects in vivo, such as reduction in food intake [54], but this suggestion requires confirmation. The concentration of B2 that is needed to observe PYY-releasing effects is higher than the concentration found in the extract. Similarly, gallic acid tended to increase PYY at a higher concentration than in GSPE, but not at that found in the active GSPE concentration. These results suggest that, in GSPE, some molecules act additively or synergically to stimulate enterohormone release. Interestingly, a mixture of catechin and epicatechin did not affect PYY duodenal secretion, but did increase CCK secretion. This, plus the fact that B2 and gallic acid did not stimulate CCK release, supports the hypothesis that a sensory machinery is activated differently by the different flavanol structures, but is also responsible for the differential secretion of enterohormones. Moreover, both catechin alone and a mixture of catechin and epicatechin increased CCK secretion. On the other hand, GSPE did not, in agreement with previous results in which GSPE at a higher concentration inhibits CCK secretion in rats [21]. In fact, we did not observe plasma CCK release after GSPE [55]. These observations suggest that compounds found in the extract antagonize the effects of catechin. We have not defined the molecules responsible for the GLP-1 stimulatory effects of GSPE, as the tested forms showed no significant effects, but a tendency towards inhibition by protocatechuic acid. It was not possible to test the effects of higher polymeric forms that may be involved in differential effects on enterohormone release. In a ghrelinoma cell line, for example, trimer C1 inhibited ghrelin secretion, which is the opposite effect to that of epicatechin gallate [22]. It has been shown that a compound can act as an agonist towards one subset of bitter receptors, but as an antagonist towards a different subset of receptors [56]. Different flavanones have been shown to antagonize epicatechin gallate response on bitter taste receptors [57] in studies that monitored calcium release in HEK293 cells expressing human bitter taste receptors. However, whether such agonism/antagonism led to differential enterohormone secretion has not been tested, though we have observed differential enterohormone secretion after stimulation with TAS2R agonists (results not shown). With ex vivo models containing not only enteroendocrine cells, but all the intestinal epithelia, we cannot ascertain the molecular mechanisms that explain this observation or discern whether there are differential mechanisms within one cell or whether there are effects on different enteroendocrine cells (Figure 4). Cellular models are used to test molecular events, though our previous results with STC-1 do not agree with enterohormone-activating secretion by GSPE or pure compounds [21,58]. Future studies will have to overcome these challenges to find out the exact mechanisms used by GSPE. Furthermore, in future studies, if the specific receptor/signalling pathway is demonstrated in pig, it will have to be established whether it is also found in humans and in which intestinal segment, as our results do not fully match between these species. In any case, these results highlight both the importance of the effects of extracts that comprise mixtures of polyphenols and the complexity involved in attributing these effects to individual molecules.

## 5. Conclusions

We have shown that GSPE stimulates basolateral enterohormone release in pig and human intestine and defined the effects of some of its components. Different flavanol structures exert different effects. B2 stimulates PYY release and catechin increases CCK, both through interaction with the apical intestinal side. These enterohormone-releasing effects are altered when flavanols are administered in combination, as occurs in natural plant extracts.

## Figures and Tables

**Table 1:** Patient characteristics.

Clinical Characteristics	Number of Patients	Percentage (%)
Gender		
Male	12	70.6
Female	5	29.4
Colon segment		
Ascending	8	47.1
Descending	9	52.9
Hypertension	11	64.7
under treatment	5	29.4
Dyslipidemia	5	29.4
under treatment	4	23.5
Diabetes mellitus type II	4	23.5
under treatment	4	23.5
**Clinical Characteristics**	**Mean ± SEM**	
Age	63.4 ± 3	
Body mass index	25.6 ± 0.8	
Blood glucose (mM)	5.8 ± 0.3	
Blood cholesterol (mg/dL)	186.6 ± 12.8	

## Supplementary Materials

The following are available online at https://www.mdpi.com/2218-273X/10/6/844/s1, Table S1: Basal enterohormone levels in the basolateral side of the controls in the different intestinal segments of pig samples.
