# Supplementary material for: Effects of Flavanols on Enteroendocrine Secretion
Source: Biomolecules. 2020 Jun 1;10(6):844. doi: 10.3390/biom10060844 (PMC7355421; doi:10.3390/biom10060844)
Supplement: Supplementary file 1 [file biomolecules-10-00844-s001.pdf]

Effects of flavanols on enteroendocrine secretion

Carme Grau; Carlos González-Quilen; Ximena Terra; MTeresa Blay; Raul Beltrán-Debón; Rosa Jorba-Martín; Beatriz Espina; Montserrat Pinent and Anna Ardévol

Supplementary materials

Table S1. Basal enterohormone levels in the basolateral side of the controls in the different intestinal segments of pig samples. Values represent mean ± SEM. * $p < 0.05$, T-test. n=7-14.

**PYY (pM)**		**GLP-1 (pM)**	
pig		pig	
duodenum	descendant colon	ascendant colon	descendant colon
0.92 ± 0.23	0.08 ± 0.16 *	10.73 ± 2.40	32.53 ± 9.80 *
**PYY (pM)**		**GLP-1 (pM)**	
human		human	
ascendant colon	descendant colon	ascendant colon	descendant colon
13.72 ± 4.09	35.85 ± 3.40 *	14.75 ± 4.27	10.73 ± 4.00
